# Medical experience as an influencing parameter in emergency medical care for psychiatric emergencies: retrospective analysis of a multicenter survey

**DOI:** 10.1186/s12873-023-00883-x

**Published:** 2023-09-23

**Authors:** Benedikt Schick, Benjamin Mayer, Constanze Hensel, Sebastian Schmid, Bettina Jungwirth, Eberhard Barth, Claus-Martin Muth, Stephan Katzenschlager, Carlos Schönfeldt-Lecuona

**Affiliations:** 1 Department of Anesthesiology and Intensive Care Medicine, University Hospital Ulm, Albert-Einstein-Allee 23, 89081 Ulm, Germany; 2 Institute of Epidemiology and Medical Biometry, Ulm University, Schwabstraße 13, 89075 Ulm, Germany; 3 Department of Anesthesiology, Heidelberg University Hospital, Im Neuenheimer Feld 420, 69120 Heidelberg, Germany; 4 Department of Psychiatry and Psychotherapy III, University Hospital Ulm, Leimgrubenweg 12–14, 89075 Ulm, Germany

**Keywords:** Prehospital emergency medicine, Psychiatric emergency, Mental disorders, Primary health care

## Abstract

**Background:**

Prehospital care of psychiatric patients often relies on the medical experience of prehospital emergency physicians (PHEPs). The psychiatrists (PSs) involved in the further treatment of psychiatric patients also often rely on their experience. Furthermore, the interaction between PHEPs and PSs is characterized by interaction problems and different approaches in the prehospital care of the psychiatric emergency.

**Objectives:**

To analyze the phenomenon of “medical experience” as a cause of possible interaction-related problems and assess its impact on the prehospital decision-making process between prehospital emergency physicians and psychiatrists.

**Methods:**

The retrospective data analysis was conducted between November 2022 and March 2023. Medical experience was defined as follows, based on the demographic information collected in the questionnaires: For PHEPs, the period since obtaining the additional qualification in emergency medicine was defined as a surrogate marker of medical experience: (i) inexperienced: < 1 year, (ii) experienced: 1–5 years, (iii) very experienced: > 5 years. For PSs, age in years was used as a surrogate parameter of medical experience: (i) inexperienced: 25–35 years, (ii) experienced: 35–45 years, (iii) very experienced: > 45 years.

**Results:**

Inexperienced PSs most frequently expressed anxiety about the psychiatric emergency referred by a PHEP (27.9%). Experienced PHEPs most frequently reported a lack of qualifications in handling the care of psychiatric emergencies (p = 0.002). Very experienced PHEPs were significantly more likely to have a referral refused by the acute psychiatric hospital if an inexperienced PS was on duty (p = 0.01). Experienced PHEPs apply an intravenous hypnotic significantly more often (almost 15%) than PSs of all experience levels (p = 0.001). In addition, very experienced PHEPs sought prehospital phone contact with acute psychiatry significantly more often (p = 0.01).

**Conclusion:**

PHEPs should be aware that the PS on duty may be inexperienced and that treating emergency patients may cause him/her anxiety. On the other hand, PHEPs should be receptive to feedback from PS who have identified a qualification deficiency in them. Jointly developed, individualized emergency plans could lead to better prehospital care for psychiatric emergency patients. Further training in the prehospital management of psychiatric disorders is needed to minimize the existing skills gap among PHEPs in the management of psychiatric disorders.

**Supplementary Information:**

The online version contains supplementary material available at 10.1186/s12873-023-00883-x.

## Background

In Germany, approximately 27% of the adult population suffers from a mental disorder each year, which corresponds to more than 17 million people [1]. In 2019, an estimated 21 million patients were treated in German Emergency Departments (German EDs), up to 10% of them with a psychiatric disorder [2]. Exact data on the prehospital care of psychiatric emergencies in Germany are missing. It is assumed that about 500,000 patients are treated by emergency physicians each year [3]. The most common psychiatric disorders of prehospital relevance are intoxication, agitation, and suicidality [3]. In Germany, prehospital patient care is provided by emergency medical services (EMS) and specialized Prehospital Emergency Physicians (PHEPs). Compared to non-psychiatric patients, people with psychiatric disorders are more likely to use emergency departments [4, 5]. In Germany, there are virtually no outpatient care structures that can treat psychiatric emergencies in the domestic setting. If a purely symptom-oriented outpatient treatment by the PHEP is not possible, then the question arises where to go. Direct referral to an acute psychiatric hospital usually fails due to interaction problems between the PHEPs and the Psychiatrists (PSs) on duty [6, 7]. Among the problems identified were a self-reported lack of PHEPs’ skills in treating psychiatric emergencies, PHEPs’ motivation to deal with psychiatric disorders, and PS’ confidence in PHEPs’ diagnostic quality [6]. Finally, the patient is referred to an emergency department, where psychiatric evaluation is usually delayed.

Another problem in the prehospital management of psychiatric emergencies is the lack of comprehensive algorithms that can be used in the treatment of psychiatric emergencies. Thus, the individual experience of the PHEP plays a much greater role than it does for patients with purely somatic disorders [3]. There is no consistent definition of “medical experience” in this context. Narrowed down to individual clinical expertise, medical experience is most likely to be the skills and judgement acquired by physicians through their training, clinical practice, and related experience. Medical behavior itself is affected to a significant extent by factors such as age, sex, cultural background, internal motivation, time restrictions, and interprofessional communication [8]. The importance of PHEPs experience as a parameter affecting the successful care of psychiatric emergencies is counteracted by the fact that an overwhelming percentage of PHEPs state that they feel inadequately qualified to handle psychiatric emergencies [6]. Therefore, the aim of the present study was to identify the impact of medical experience on interaction problems between PHEPs and PSs in the prehospital treatment of psychiatric emergencies and to ascertain the impact of medical experience on prehospital approaches of PHEPs and PSs in the management of psychiatric emergencies based on a casuistry.

## Methods

### Study design

A prospective, questionnaire-based, multicenter, anonymized survey was conducted at five maximum care and one primary care hospital as well as five psychiatric maximum care and seven primary care hospitals in Germany between March and October 2021 [6]. The participating hospitals were specifically selected from the authors’ personal network, since these centers are intensively cooperating in clinical issues concerning the care of patients with psychiatric disorders. This secondary data analysis was performed after the publication of the prospective data in October 2022 and covered the period between November 2022 and March 2023.The questions were designed in such a way that PSs had to assume the role of the PHEP in some respects. The study was designed by the Department of Anesthesiology and Intensive Care Medicine and the Department of Psychiatry and Psychotherapy III at the University Hospital Ulm. We submitted our work to the local ethics committee (Ethics Committee of the University of Ulm, Germany) for evaluation. The local ethics committee reviewed the formalities of the study; as this was an anonymous survey of medical professionals with retrospective character, no specific ethical approval was needed.

### Characteristics of the questionnaires

The questionnaires were designed by an anesthetist actively practicing prehospital emergency medicine and an experienced psychiatrist and is described in detail in [6]: After an internal pretest phase and subsequent adjustment, the questionnaires were approved for use. The questionnaires were divided into three sections (see supplement): (i) in the first part, questions were related to respondents’ personal rating of the keyword “psychiatric” (Questions 1 to 2a). Possible structural problems in emergency medical care were also addressed in this section; (ii) in the second section, a typical case that would arise as part of the everyday work of an PHEP was presented on the basis of a case vignette in order to determine the emergency medical procedure and possible problem-solving strategies (Questions 3 to 5); (iii) the third section of the questionnaire dealt with the need for improved training and continuing education opportunities relating to clinical psychiatric conditions (Questions 6 to 7). Demographic data were collected at the end of the questionnaires. The questionnaires were distributed by contact persons in the respective hospitals and returned using the return boxes provided or directly to the authors. Participating hospitals received reminders to complete the surveys at the two- and four-month marks, which explains the long survey period. The return rate of the questionnaires varied between 12% and 84% because each hospital initially received 20 questionnaires, regardless of the number of potential participants. Prerequisites for participation in the survey included active participation in prehospital emergency medical services or regular shifts in a ward with acute psychiatric admissions [6]. The questionnaires are included in the supplement.

### Study objectives

The primary objective of the study was to investigate whether:

- medical experience affects interaction-related problems between PHEPs and PSs in the treatment of psychiatric emergencies.
- the physician’s level of experience affects the prehospital problem-solving behavior of the PHEP and the PS as well as the elements of an individualized emergency plan for the psychiatric emergency.

The secondary objective of the study was to investigate whether:

- medical experience leads to a different perception of the training needs of PHEPs and PSs in the care of psychiatric emergencies.

#### Definition of “Medical experience”

Medical experience was defined as follows, based on the demographic information collected in the questionnaires:

For PHEPs, the period since obtaining the additional qualification in emergency medicine was defined as a surrogate marker of medical experience: (i) inexperienced: < 1 year, (ii) experienced: 1–5 years, (iii) very experienced: > 5 years.

Different criteria apply for the additional qualification in emergency medicine in Germany. The following are the minimum requirements that must be met [9]:

- 24 months of further training in an area of direct patient care in an inpatient setting, including 6 months in intensive care or anesthesia or in an interdisciplinary central emergency department.
- In addition, an 80-hour training course in general and specialized emergency care must be completed, followed by 50 emergency medical missions (ambulance or helicopter) supervised by a board-certified emergency physician.

There is no further supervision if the additional qualification in emergency medicine has been obtained. Periodic training in emergency medicine is not required.

For PSs, age in years was used as a surrogate parameter of medical experience: (i) inexperienced: 25–35 years, (ii) experienced: 35–45 years, (iii) very experienced: > 45 years. The classification of psychiatrists’ experience was based on the average age of university graduates in human medicine, which was 26.1 years in Germany in 2021 [10]. Specialist training to become a psychiatrist takes at least 60 months. On average, therefore, most psychiatrists will be at least 32 years old when they become specialists in their field. The age corridors selected are therefore close to the medical experience of the psychiatrists surveyed.

**Fig. 1:**
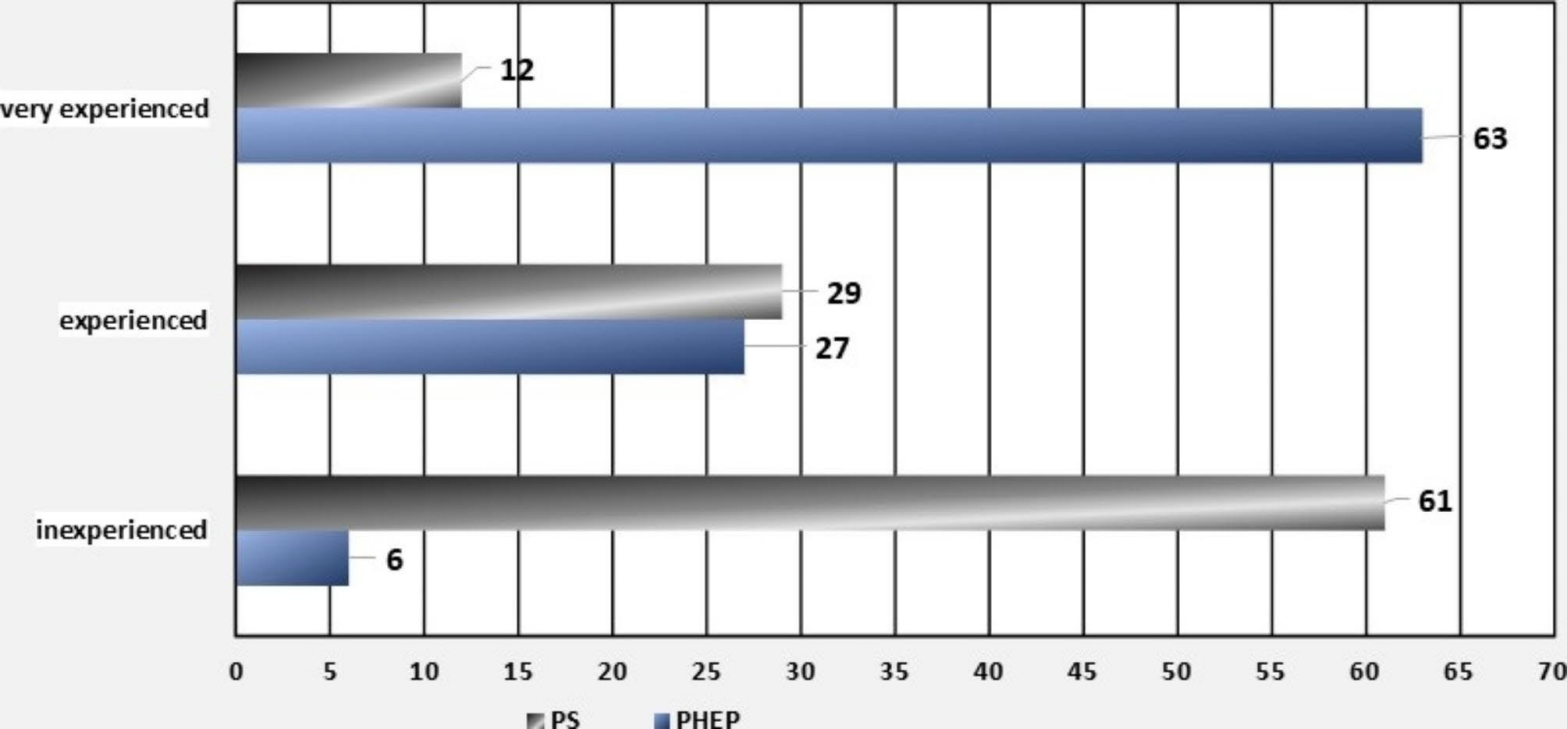
**Overview of the prehospital emergency physicians (PHEPs) and psychiatrists (PSs) interviewed, based on their experience.** The graph shows prehospital emergency physicians (blue bars) and psychiatrists (grey bars) from bottom to top, starting with the inexperienced PHEPs (< 1 year of additional qualification in emergency medicine) and the inexperienced PSs (25–35 years), followed by the experienced PHEPs (1–5 years of additional qualification in emergency medicine) and the experienced PSs (35–45 years) as well as the very experienced PHEPs (> 5 years of additional qualification in emergency medicine) and the very experienced PSs (> 45 years). The number of respondents can be read on the abscissa. PHEP: prehospital emergency physician, PS: psychiatrist

Figure 1 demonstrates the distribution of experience across both specialties. The very broad time periods for the time since obtaining the additional qualification in emergency medicine for PHEPs and the age for PSs were deliberately chosen in order to ensure the anonymity of the survey.

All of the PHEPS respondents were anesthetists. 1 respondent also reported being a specialist in internal medicine. Another respondent was also a specialist in neurology.

None of the psychiatrists interviewed reported an additional specialty.

#### Definition of interaction problems and prehospital problem-solving behavior of PHEPs and PSs

The data refer to the publication of the prospective data set [6] and are defined as follows:

**Interaction problems between PHEPs and PSs**:

- Feeling of being insufficiently qualified to handle psychiatric emergencies.
- Anxiety about the psychiatric emergency.
- Reasons for refusal of hospital admission for the psychiatric emergency.

**Prehospital problem-solving behaviors and elements of an individualized treatment protocol**:

- Administration of medication (application method: oral/IV/MAD/, dosage, time of administration).
- Criteria for outpatient or inpatient care of the psychiatric emergency.
- Seeking telephone contact with the acute psychiatric hospital.

### Statistical analysis

The responses to the questionnaires were recorded using Microsoft EXCEL 2021® (Microsoft Corp., Redmond, WA). The statistical analyses were performed with Sigma Plot Version 14® for Windows (Systat Software GmbH, Erkrath, Germany) and SPSS Version 28® (Statistical Package for Social Science, IBM, Armonk-New York, USA). The descriptive analysis of the questionnaire characteristics differed depending on the type of variable and was done by means of frequencies and percentages for categorical characteristics or based on the arithmetic mean, standard deviation (SD), median, and range for metrically scaled characteristics. Further analysis of possible differences between the two groups of PHEPs and PSs was performed using appropriate exploratory hypothesis tests, for which a two-sided type 1 error rate of 5% was assumed. The chi-square test was used for multiple response sets for categorical endpoints. Metrically scaled endpoints were evaluated with either the unpaired t-test or the Mann-Whitney U-test after checking the normal distribution assumption using the Shapiro-Wilk test. The p-values in Table 1, as well as all other p-values throughout the paper, are to be interpreted in a fully explorative manner. Due to the retrospective nature of the presented study, confirmatory hypothesis testing is not possible, i.e. an adjustment of p-values is not reasonable.

## Results

**Fig. 2:**
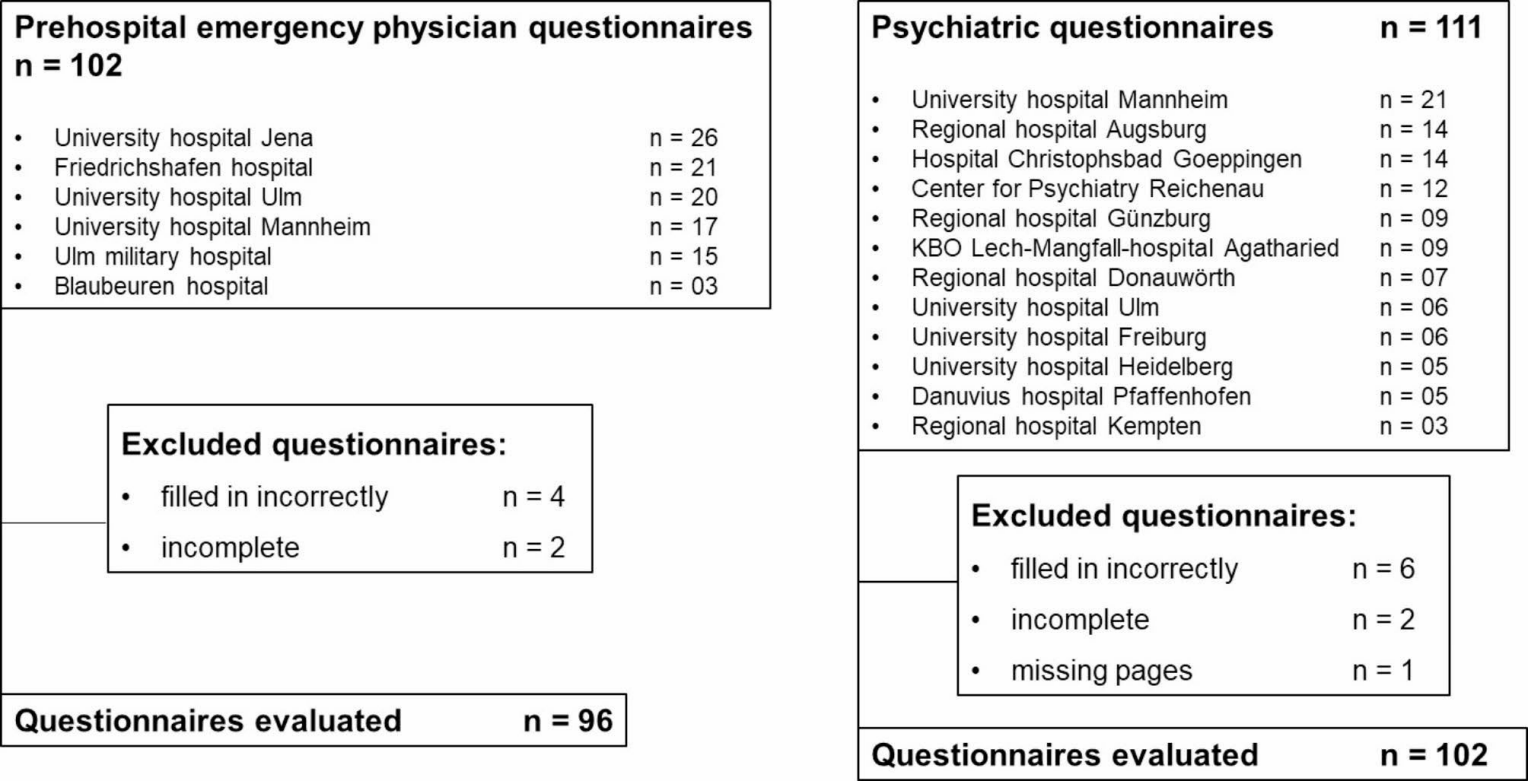
**Study flowchart**. This figure provides an overview of the participating hospitals, differentiated by prehospital emergency physician location and psychiatric treatment facilities, and also takes into consideration the questionnaires excluded

Figure 2 provides an overview of the hospitals participating in the survey. For further information about the participating hospitals see supplementary tables s2 and s3.

As can be seen in Table 1, neither the experience of the PHEPs nor that of the PSs had an influence on the interaction problems identified or the prehospital problem-solving behavior in the case vignette described. The results are reported based on the questions and answers given in the questionnaires.

**Table 1:** **Results of the Multiple Set analysis for the effect of “medical experience.“** The statistical differences within the PHEP group (inexperienced, experienced, very experienced) and within the PS group (inexperienced, experienced, very experienced) were calculated by means of pairwise chi-square tests. PHEP: Prehospital emergency physician, PS: Psychiatrist, Q: Question. For detailed information on the underlying answers, see [6]

	PHEP experience p-value	PS experience p-value
Q_1_: Rating of the emergency call “psychiatric emergency.“	0.24	0.19
**Q**_**2**_: **Reasons for refusal of hospital admission.**	0.64	0.96
**Q**_**3**_: **Casuistry**: **Post-traumatic stress disorder, agitation, hyperventilation, verbal calming not possible.**	0.13	0.98
**Q**_**3a**_: **Different options for medication application in the casuistry/psychiatric emergency.**	0.66	0.47
**Q**_**4**_: **Possible elements of a treatment protocol for the psychiatric emergency.**	0.64	0.43
**Q**_**7**_: **Requirement for further training and information for emergency physicians.**	0.80	0.06

### Interaction problems between PHEPs and PSs depending on medical experience

#### Feeling of insufficient qualification for psychiatric emergencies

Of all respondents, experienced PHEPs were the most likely to report that they do not feel sufficiently qualified to deal with psychiatric emergencies (n = 11, 40.7%), especially in comparison with the inexperienced and experienced PSs (PS_inexperienced_: n = 6, 9.8%, PS_experienced_: n = 5, 17.2%, p = 0.002). Inexperienced PHEPs also reported feeling a qualification deficit with regard to handling the psychiatric emergency significantly more often than their equally inexperienced psychiatric colleagues (PHEP_inexperienced_: n = 3, 50% vs. PS_inexperienced_: n = 6, 9.8%, p = 0.03). More very experienced PSs addressed a deficiency in qualifications for handling psychiatric emergencies than very experienced PHEPs (PHEP_very experienced_: n = 4, 6.3% vs. PS_experienced_: n = 5, 17%, p = 0.03). For further comparisons between PHEPs and PSs, see Table 1 – Supplement.

#### Anxiety about the psychiatric emergency

Anxiety around psychiatric emergencies referred by the PHEP was most likely to be reported by inexperienced PSs (PS_inexperienced_: n = 17, 27.9% vs. PHEP_inexperienced_: n = 0, p = 0.32; vs. PHEP_experienced_: n = 2, 7.4%, p < 0.001). For further comparisons between PHEPs and PSs, see Table 1 – Supplement.

#### Reasons for refusal of hospital admission for the psychiatric emergency

The likelihood of a very experienced PHEP being refused an admission to the psychiatric emergency department was significantly higher when an inexperienced PS was on duty (PHEP_very experienced_: n = 53 vs. PS_inexperienced_: n = 47, p = 0.01). Reasons for refusal included a lack of bed capacity (p = 0.09) and the assumption that the catchment area of the hospital was justified as a reason for refusal (p = 0.004). For further comparisons between PHEPs and PSs, see Table 1 – Supplement.

### Prehospital problem-solving behaviors and elements of an individualized treatment protocol

#### Administration of medication (application method: oral/intravenous/nasal/intra muscular, dosage, time of administration)

Most often, experienced PHEPs would recommend intravenous (IV) administration of a hypnotic as an alternative to oral benzodiazepine for a patient with dissociative disorder (n = 4, 14.8%), while this would not be an option for any of the PSs (p = 0.001). Experienced and very experienced PHEPs would also use a mucosal atomization device (MAD) for nasal application more often than their psychiatric colleagues. In the case of the dissociative disorder described in the casuistry, the prehospital administration of intramuscular medication (IM) plays only a minor role for the PHEPs. PSs would consider the use of IM with a similar frequency as the above-mentioned MAD (see Supplementary table s1).

#### Seeking telephone contact with the acute psychiatric hospital

Very experienced PHEPs would consider seeking prehospital telephone contact with the respective psychiatric hospital significantly more often than experienced PSs in the same situation (PHEP_very experienced_: n = 54, 85.7%, PS_experienced_: n = 21, 72.4%, p = 0.01). For further comparisons of the different medical experience levels, see Table 1 – Supplement.

#### Elements of a treatment protocol for the psychiatric emergency

As shown in Supplementary table s1, experienced PHEPs were more concerned about the elements of the treatment protocol than experienced or very experienced PSs. Like PSs, PHEPs rated an individualized emergency concept for acute psychiatric patients with recurrent contact to the PHEP as useful, irrespective of their experience. When asked about the need for further training on the topic of psychiatric emergencies, 100% of the inexperienced PHEPs (N = 6) stated that they would like to have more training on typical prehospital acute psychiatric clinical pictures as well as legal principles regarding the hospitalization of patients. Only about 70% (N = 19) of experienced PHEPs and about 80% (N = 49) of very experienced PHEPs wanted more training. When PSs were asked how they perceived the need for further training for PHEPs regarding the prehospital care of psychiatric emergencies, 95% of the inexperienced PSs (N = 58) and 100% of the experienced PSs (N = 29) stated that they would consider further training in this area necessary. Among the very experienced PSs, 75% (N = 9) still felt that PHEPs needed more training in the care of psychiatric emergencies. PSs were also asked whether they would like to have a better insight into emergency medicine, for example in the context of a one-day internship with the ambulance. About 75% of the psychiatrists, regardless of their experience, would take advantage of such an offer (see Supplementary table s1).

## Discussion

This study showed that the medical experience of PHEPs and PSs influences the interaction problems between PHEPs and PSs in the prehospital care of psychiatric emergencies.

### Anxiety and insufficient qualifications as a problem identified as affecting interaction between PHEPs and PSs

In the medical care of emergency patients, especially in psychiatric emergencies (most often intoxications and/or states of agitation of any kind), the risk of becoming a victim of verbal or physical violence is significantly increased [11–14]. As a survey by the German Association for Psychiatry, Psychotherapy and Psychosomatics (DGPPN) revealed in 2016, inexperienced staff are particularly at risk [15]. Therefore, at first glance, it does not seem surprising that the inexperienced PSs stated that they were afraid of acute psychiatric patients admitted by PHEPs or of the responsibility and circumstances this entailed. Anxiety leads to an undesirable stress reaction in the person concerned. If the stress level exceeds a moderate, performance-enhancing range, the ability to judge and act suffers which in turn endangers patient safety and can perpetuate the fears of the individual in the sense of a self-reinforcing model. Causes for the fear of colleagues could include the lack of qualification addressed by some respondents, but also the fact that predominantly inexperienced colleagues must take responsibility for and manage the admission of seriously ill, sometimes aggressive patients. PHEPs should take this into account when considering whether a psychiatric emergency should be assigned to an acute psychiatric hospital. In particular, possible somatic concomitant disorders as well as disorders requiring observation (e.g., severe mixed intoxications) should be evaluated critically and, if necessary, first transferred to a central emergency department for “medical clearing” [3, 6, 16].

By contrast, only a very small percentage of PHEPs stated that they were afraid of psychiatric emergencies. It is possible that PHEPs have a higher sense of security because they work in a team, usually consisting of 4–5 people (PHEPs and paramedics). The fact that experience cannot be the only determining factor in the evaluation of the inexperienced PSs` fear is also shown in a survey of internal medicine specialists on the second victim phenomenon by Strametz et al. Medical experience (> 6 years) was shown to be a risk factor for the traumatization of healthcare staff themselves by a stressful event in the context of patient care [17].

In contrast to the internalized uncertainty of the inexperienced PSs, experienced PHEPs most frequently stated that they felt insufficiently qualified to handle psychiatric emergencies. Pajonk et al. already identified a possible deficit in the qualification of German PHEPs for the treatment of psychiatric emergency patients some 20 years ago [18]. To improve their skills in the treatment of psychiatric emergencies, PHEPs stated in a survey that they were willing to complete a one-day internship in a psychiatric hospital [6]. In addition, psychiatric emergency topics for which more training was needed were clearly identified by emergency physicians. These included dissociative seizures, intoxication, suicidality, and legal aspects of dealing with psychiatric patients. Training concepts to improve the quality of prehospital care for psychiatric patients should specifically address these aspects, but also take into account local aspects of infrastructure and collaboration between PSs and PHEPs [3].

The problem of lack of qualification to treat psychiatric emergencies is not just a problem among PHEPs, but also among paramedics. In a study of Australian paramedics with more than 5 years of professional experience, Roberts et al. were able to demonstrate that almost 42% of the respondents also felt inadequately trained to treat psychiatric emergencies. Among paramedics with less than 5 years of professional experience, the rate was as high as 62% [19]. The underlying reasons are similar, even though the emergency medical systems are organized in completely different ways. First and foremost, there is a lack of properly organized education and training on the prehospital treatment of psychiatric emergencies. Shirzad et al. attempted to use a protocol-based approach to organize prehospital paramedic-based care for psychiatrically ill patients [20]. This appears appropriate considering the uncertainties pertaining to this group of patients. However, protocols by their very nature do not consider the individual needs of patients. Individualized emergency plans, which achieved a high level of acceptance in a survey of psychiatrists and emergency physicians, are more effective in achieving this [6].

### Individualized emergency plans

Individualized emergency plans for patients with psychiatric disorders are quite rare in their prehospital management. Access to information about psychiatrically ill patients with recurrent contact to prehospital medical care structures may enable PHEPs/rescue service staff to apply diagnostic and therapeutic measures in a targeted manner and may help to reduce these to the necessary extent, and may eventually reduce the risk of overtreatment. Quinn et al. attempted to analyze the requirements and the necessary content for an emergency ID card for psychiatric patients. They concluded that, among other things, the kind of disorder, the associated symptoms, and helpful behavior, medication and possible allergies should be documented as bullet points [21]. These items are essentially in line with what PHEPs and PSs consider important information in an individualized emergency plan for selected psychiatrically ill patients and achieved a very high acceptance level of > 90% in a survey [6]. Because emergency patients often carry a cell phone, psychiatric patients should be made aware that some manufacturers offer the ability to store emergency documents that emergency responders can access without a security code.

### Medical experience as an aspect of interdisciplinary communication

At the time of the survey, inexperienced PSs were unlikely to be board certified in psychiatry. Although it is not known, it can be assumed that they are often not allowed to accept emergency patients until they have consulted with a senior physician. A survey of hospital physicians published in 2020 found that communication among physicians was rated negatively, especially in university hospitals [22]. It is possible that such a negative communication structure within a medical department also influences communication with external parties, in this case PHEPs. It is also conceivable that inexperienced PSs may have had negative contact with PHEPs in the past. A negative communication experience may have a negative impact on future communication in terms of a negativity bias, i.e., the tendency to remember bad experiences, and may also increase feelings of anxiety [23, 24]. The negativity bias is most pronounced among younger people. This, in turn, could be indirectly transferred to inexperienced PSs, for whom problems in interacting with PHEPs could be most frequently demonstrated [25].

### Limitations of the study

Due to its questionnaire-based, anonymous design, the study is subject to a number of limitations that need to be discussed at this point. The classification of PHEPs and PSs into different levels of experience that are not directly comparable is the primary weakness of the study. A prospective study with a definition that allows comparability of medical experience between PHEPs and PSs would be needed to eliminate this bias.

Demographic data such as exact age and sex, which are potential confounders for the influencing factor “medical experience,“ were not collected a priori. After clustering the data set into subgroups, the inexperienced and very experienced PHEPs, as well as the very experienced PSs, had very small case numbers, which hindered meaningful statistical analysis of the data and allowed only partial conclusions to be drawn. In addition to the additional qualification in emergency medicine, which all PHEPs interviewed had, many anesthetists have other qualifications such as in intensive care, pain- or palliative medicine. This, in turn, may influence the respondents’ prehospital management of psychiatric emergencies. The criteria for obtaining the additional qualification in emergency medicine do not differ in principle between university hospitals and smaller hospitals due to centrally organized training courses and exams. Therefore, according to the current evidence, it can be assumed that the qualification deficit of PHEPs in the treatment of psychiatric patients, which has already been addressed, exists independently of their experience [6, 7, 18]. The level of training of the psychiatrists surveyed in the participating psychiatric hospitals is not known. Most of the psychiatrists surveyed were 35 years of age or younger, suggesting that they may have been predominantly inexperienced. Similar to anesthesiology, psychiatrists may have other additional qualifications that have an impact on both patient care and interaction with PHEPs.

PHEPs were almost exclusively anesthetists. Especially in rural facilities within the Federal Republic of Germany, physicians from other specialties often practice emergency medicine. Accordingly, the significance of the results is limited and they should primarily be interpreted in the context of an anesthesiological/emergency medical approach to care. Furthermore, the emergency medical assistance service as it is organized in Germany cannot be applied to other countries. In view of the increasing shift in competence towards emergency paramedics as well as the implementation of tele-PHEP services, the way in which psychiatric emergencies will be treated in a prehospital setting in the future remains unclear [26].

### Conclusion

In conclusion, psychiatric emergencies are challenging for both PHEPs and PSs. In this context, PHEPs should be aware that the further treatment of psychiatric emergency patients is often performed by a comparatively inexperienced colleague who may be anxious about the patient and the associated situation. A patient ID card containing key information about the patient’s psychiatric disease could be a valuable resource for the prehospital setting, since the acceptance of individualized emergency plans is very high among both PHEPs and PSs, regardless of their level of medical experience. Further training in the prehospital management of psychiatric disorders is needed to minimize the existing skills gap among PHEPs in the management of psychiatric disorders.

### Electronic supplementary material

Below is the link to the electronic supplementary material.

Supplementary Material 1

## Data Availability

The data sets created and/or analyzed as part of this study are not publicly available, as some information is used for internal quality analysis only. All other results and data collected are reported entirely within the scope of this manuscript. If there is a justified interest in accessing it, the data can be requested from the corresponding author.

## Abbreviations

DGPPN: German Association for Psychiatry, Psychotherapy and Psychosomatics
ED: Emergency Department
MAD: Mucosal Atomization Device
PHEP: Prehospital Emergency Physician
PS: Psychiatrist
