# Supplementary material for: Medical experience as an influencing parameter in emergency medical care for psychiatric emergencies: retrospective analysis of a multicenter survey
Source: BMC Emerg Med. 2023 Sep 23;23:112. doi: 10.1186/s12873-023-00883-x (PMC10517561; doi:10.1186/s12873-023-00883-x)
Supplement: Supplementary file 1 — Supplementary Material 1 [file 12873_2023_883_MOESM1_ESM.docx]

**Medical experience as an influencing parameter in emergency medical care of psychiatric emergencies: Retrospective analysis of a multicentre survey**

Benedikt Schick^1*^, Benjamin Mayer^2^, Constanze Hensel^1^, Sebastian Schmid^1^, Bettina Jungwirth^1^, Eberhard Barth^1^, Claus-Martin Muth^1^, Stephan Katzenschlager³, Carlos Schönfeldt-Lecuona^4^

**Supplement**

| **Question 1 – Rating of the emergency call "psychiatric emergency"** | | | | |
| --- | --- | --- | --- | --- |
|  | PHEP_inexp._  (n=6) | PS_inexp._ (n=61) | PS_experienced_  (n=29) | PS_very hexp._  (n=12) |
|  |  | 0.01 | 0.05 | 0.15 |
| Rating of the psychiatric emergency as “meaningless” or as “incorrect diagnosis, made by the EM” | 0 | 27 (44.2%) | 13 (44.8%) | 3 (25%) |
| Feeling of insufficient qualification for psychiatric emergencies | 3 (50%) | 6 (9.8%) | 5 (17.2%) | 1 (8.3%) |
| Anxious about the psychiatric emergency | 0 | 17 (27.9%) | 2 (6.9%) | 1 (8.3%) |
| Making no difference between psychiatric emergencies and other emergencies | 3 (50%) | 31 (50.8%) | 19 65.5%) | 8 (66.7%) |
|  | PHEP_exp._  (n=27) | PSi_nexp._ (n=61) | PS_experienced_  (n=29) | PS_very hexp._  (n=12) |
|  |  | 0.003 | 0.02 | 0.08 |
| Rating of the psychiatric emergency as “meaningless” or as “incorrect diagnosis, made by the EM” | 12 (44.4%) | 27 (44.2%) | 13 (44.8%) | 3 (25%) |
| Feeling of insufficient qualification for psychiatric emergencies | 11 (40.7%) | 6 (9.8%) | 5 (17.2%) | 1 (8.3%) |
| Anxious about the psychiatric emergency | 2 (7.4%) | 17 (27.9%) | 2 (6.9%) | 1 (8.3%) |
| Making no difference between psychiatric emergencies and other emergencies | 10 (37.0%) | 31 (50.8%) | 19 (65.5%) | 8 (66.7%) |
|  | PHEP_very exp._ (n=63) | PS_inexp._ (n=61) | PS_experienced_  (n=29) | PS_very hexp._  (n=12) |
|  |  | <0.001 | 0.01 | 0.19 |
| Rating of the psychiatric emergency as “meaningless” or as “incorrect diagnosis, made by the EM” | 16 (25.4%) | 27 (44.2%) | 13 (44.8%) | 3 (25%) |
| Feeling of insufficient qualification for psychiatric emergencies | 4 (6.3%) | 6 (9.8%) | 5 (17.2%) | 1 (8.3%) |
| Anxious about the psychiatric emergency | 0 | 17 (27.9%) | 2 (6.9%) | 1 (8.3%) |
| Making no difference between psychiatric emergencies and other emergencies | 32 (50.8%) | 31 (50.8%) | 19 65.5%) | 8 (66.7%) |
| **Question 2 - Reasons for refusal of hospital admission** | | | | |
|  | PHEPi_nexp._  (n=4) | PSi_nexp._ (n=47) | PS_experienced_  (n=27) | PS_very hexp._  (n=10) |
|  |  | 0.47 | 0.57 | 0.89 |
| No capacity | 2 (50%) | 11 (23.4%) | 4 (14.8%) | 4 (40%) |
| Intoxication of the patient | 3 (75%) | 43 (91.5%) | 23 (85.2%) | 10 (100%) |
| Preclinically applied medication | 2 (50%) | 29 (61.7%) | 18 (66.7%) | 6 (60%) |
| Patient not assigned to the hospitals catchment area | 2 (50%) | 23 (48.9%) | 13 (48.2%) | 5 (50%) |
|  | PHEP_exp._  (n=21) | PSi_nexp._ (n=47) | PS_experienced._  (n=27) | PS_very exp._  (n=10) |
|  |  | 0.13 | 0.32 | 0.57 |
| No capacity | 5 (23.8%) | 11 (23.4%) | 4 (14.8%) | 4 (40%) |
| Intoxication of the patient | 16 (76.2%) | 43 (91.5%) | 23 (85.2%) | 10 (100%) |
| Preclinically applied medication | 11 (52.4%) | 29 (61.7%) | 18 (66.7%) | 6 (60%) |
| Patient not assigned to the hospitals catchment area | 12 (57.1%) | 23 (48.9%) | 13 (48.2%) | 5 (50%) |
|  | PHEP_very exp_ (n=53) | PS_inexp._ (n=47) | PS_high exp._  (n=27) | PS_very high exp._  (n=10) |
|  |  | 0.01 | 0.07 | 0.34 |
| No capacity | 21 (39.6%) | 11 (23.4%) | 4 (14.8%) | 4 (40%) |
| Intoxication of the patient | 44 (83.0%) | 43 (91.5%) | 23 (85.2%) | 10 (100%) |
| Preclinically applied medication | 30 (56.6%) | 29 (61.7%) | 18 (66.7%) | 6 (60%) |
| Patient not assigned to the hospitals catchment area | 40 (75.5%) | 23 (48.9%) | 13 (48.2%) | 5 (50%) |
| **Question 3 - Casuistry: post-traumatic stress disorder, agitation, hyperventilation, no verbal calming possible.** | | | | |
|  | PHEP_inexp._  (n=6) | PS_inexp_ (n=61) | PS_experienced_  (n=29) | PS_very hexp._  (n=12) |
|  |  | 0.02 | 0.20 | 0.14 |
| Talk down technique | 6 (100%) | 57 (93.4%) | 27 (93.1%) | 11 (91.7%) |
| Benzodiazepine administration | 3 (50%) | 47 (77.1%) | 23 (79.3%) | 10 (83.3%) |
| Hypnotic administration (e.g. propofol) | 1 (16.7%) | 0 | 0 | 0 |
| Antipsychotic administration (e.g. haloperidol) | 1 (16.7%) | 3 (4.9%) | 2 (6.9%) | 0 |
| Involving the police to obtain psychiatric admission | 0 | 2 (3.2%) | 2 (6.9%) | 0 |
| Seek phone contact with the acute psychiatric hospital | 5 (83.3%) | 36 (59%) | 21 (72.4%) | 6 (50%) |
| Abandoning all further attempts to ensure admission | 0 | 4 (6.6%) | 2 (6.9%) | 1 (8.3%) |
|  | PHEP_exp_ (n=27) | PS_inexp_ (n=61) | PS_experienced_  (n=29) | PS_very hexp._  (n=12) |
|  |  | 0.14 | 0.20 | 0.20 |
| Talk down technique | 25 (92.6%) | 57 (93.4%) | 27 (93.1%) | 11 (91.7%) |
| Benzodiazepine administration | 23 (85.2%) | 47 (77.1%) | 23 (79.3%) | 10 (83.3%) |
| Hypnotic administration (e.g. propofol) | 4 (14.8%) | 0 | 0 | 0 |
| Antipsychotic administration (e.g. haloperidol) | 0 | 3 (4.9%) | 2 (6.9%) | 0 |
| Involving the police to obtain psychiatric admission | 1 (3.7%) | 2 (3.2%) | 2 (6.9%) | 0 |
| Seek phone contact with the acute psychiatric hospital | 23 (85.2%) | 36 (59%) | 21 (72.4%) | 6 (50%) |
| Abandoning all further attempts to ensure admission | 0 | 4 (6.6%) | 2 (6.9%) | 1 (8.3%) |
|  | PHEP_very exp_ (n=63) | PS_inexp_ (n=61) | PS_experienced_  (n=29) | PS_very hexp._  (n=12) |
|  |  | 0.01 | 0.28 | 0.08 |
| Talk down technique | 58 (92.1%) | 57 (93.4%) | 27 (93.1%) | 11 (91.7%) |
| Benzodiazepine administration | 42 (66.7%) | 47 (77.1%) | 23 (79.3%) | 10 (83.3%) |
| Hypnotic administration (e.g. propofol) | 1 (1.6%) | 0 | 0 | 0 |
| Antipsychotic administration (e.g. haloperidol) | 1 (1.6%) | 3 (4.9%) | 2 (6.9%) | 0 |
| Involving the police to obtain psychiatric admission | 6 (9.5%) | 2 (3.2%) | 2 (6.9%) | 0 |
| Seek phone contact with the acute psychiatric hospital | 54 (85.7%) | 36 (59%) | 21 (72.4%) | 6 (50%) |
| Abandoning all further attempts to ensure admission | 1 (1.6%) | 4 (6.6%) | 2 (6.9%) | 1 (8.3%) |
| **Question 3a - Different options of medication application in the Casuistry/psychiatric emergency** | | | | |
|  | PHEP_inexp._  (n=6) | PS_inexp_ (n=61) | PS_experienced_  (n=29) | PS_very hexp._  (n=12) |
|  |  | <0.001 | <0.001 | 0.14 |
| Intra venous | 3 (50%) | 4 (6.6%) | 0 | 0 |
| Intra osseous | 0 | 0 | 0 | 0 |
| Mucosal Atomization Device | 2 (33.3%) | 6 (9.8%) | 3 (10.3%) | 2 (16.7%) |
| Intra muscular | 1 (16.7%) | 6 (9.8%) | 2 (6.9%) | 1 (8.3%) |
| Oral drug administration | 2 (33.3%) | 57 (93.4%) | 27 (93.1%) | 10 (83.3%) |
| None of the options mentioned above | 0 | 3 (4.9%) | 1 (3.4%) | 2 (16.7%) |
|  | PHEP_exp_ (n=27) | PS_inexp_ (n=61) | PS_experienced_  (n=29) | PS_very hexp._  (n=12) |
|  |  | <0.001 | <0.001 | 0.04 |
| Intra venous | 9 (33.3%) | 4 (6.6%) | 0 | 0 |
| Intra osseous | 0 | 0 | 0 | 0 |
| Mucosal Atomization Device | 10 (37%) | 6 (9.8%) | 3 (10.3%) | 2 (16.7%) |
| Intra muscular | 1 (3.7%) | 6 (9.8%) | 2 (6.9%) | 1 (8.3%) |
| Oral drug administration | 20 (74.1%) | 57 (93.4%) | 27 (93.1%) | 10 (83.3%) |
| None of the options mentioned above | 0 | 3 (4.9%) | 1 (3.4%) | 2 (16.7%) |
|  | PHEP_very exp_ (n=63) | PS_inexp_ (n=61) | PS_experienced_  (n=29) | PS_very hexp._  (n=12) |
|  |  | <0.001 | <0.001 | 0.02 |
| Intra venous | 25 (39.7%) | 4 (6.6%) | 0 | 0 |
| Intra osseous | 0 | 0 | 0 | 0 |
| Mucosal Atomization Device | 24 (38.1%) | 6 (9.8%) | 3 (10.3%) | 2 (16.7%) |
| Intra muscular | 3 (4.8%) | 6 (9.8%) | 2 (6.9%) | 1 (8.3%) |
| Oral drug administration | 44 (69.8%) | 57 (93.4%) | 27 (93.1%) | 10 (83.3%) |
| None of the options mentioned above | 2 (3.2%) | 3 (4.9%) | 1 (3.4%) | 2 (16.7%) |
| **Question 4 - Possible items of a treatment protocol for the psychiatric emergency** | | | | |
|  | PHEP_inexp._  (n=6) | PS_inexp_ (n=61) | PS_experienced_  (n=29) | PS_very hexp._  (n=12) |
|  |  | 0.71 | 0.59 | 0.59 |
| Possible symptoms of the dissociative seizure | 6 (100%) | 47 (77.1%) | 20 (69.0%) | 9 (75%) |
| The expected duration of the seizure | 4 (66.7%) | 38 (62.3%) | 17 (58.6%) | 5 (41.7%) |
| Helpful behavior | 6 (100%) | 59 (96.7%) | 27 (93.1%) | 12 (100%) |
| The point at which medical therapy should be considered | 3 (50%) | 43 (70.5%) | 16 (55.2%) | 7 (58.3%) |
| The type and dosage of the drug to be used | 6 (100%) | 48 (78.7%) | 21 (72.4%) | 10 (83.3%) |
| Circumstances under which outpatient care would be possible | 5 (83.3%) | 44 (72.1%) | 18 (62.1%) | 6 (50%) |
| Conditions for inpatient admission | 5 (83.3%) | 56 (91.8%) | 23 (79.3%) | 10 (83.3%) |
| Question cannot be answered | 0 | 1 (1.6%) | 1 (3.4%) | 0 |
|  | PHEP_exp_ (n=27) | PS_inexp_ (n=61) | PS_experienced_  (n=29) | PS_very hexp._  (n=12) |
|  |  | 0.43 | 0.01 | 0.003 |
| Possible symptoms of the dissociative seizure | 24 (88.9%) | 47 (77.1%) | 20 (69.0%) | 9 (75%) |
| The expected duration of the seizure | 19 (70.4%) | 38 (62.3%) | 17 (58.6%) | 5 (41.7%) |
| Helpful behavior | 25 (92.6%) | 59 (96.7%) | 27 (93.1%) | 12 (100%) |
| The point at which medical therapy should be considered | 24 (88.9%) | 43 (70.5%) | 16 (55.2%) | 7 (58.3%) |
| The type and dosage of the drug to be used | 23 (85.2%) | 48 (78.7%) | 21 (72.4%) | 10 (83.3%) |
| Circumstances under which outpatient care would be possible | 27 (100%) | 44 (72.1%) | 18 (62.1%) | 6 (50%) |
| Conditions for inpatient admission | 25 (92.6%) | 56 (91.8%) | 23 (79.3%) | 10 (83.3%) |
| Question cannot be answered | 0 | 1 (1.6%) | 1 (3.4%) | 0 |
|  | PHEP_very exp_ (n=63) | PSi_nexp_ (n=61) | PS_experienced_  (n=29) | PS_very hexp._  (n=12) |
|  |  | 0.12 | 0.003 | 0.03 |
| Possible symptoms of the dissociative seizure | 50 (79.4%) | 47 (77.1%) | 20 (69.0%) | 9 (75%) |
| The expected duration of the seizure | 42 (66.7%) | 38 (62.3%) | 17 (58.6%) | 5 (41.7%) |
| Helpful behavior | 58 (92.1%) | 59 (96.7%) | 27 (93.1%) | 12 (100%) |
| The point at which medical therapy should be considered | 45 (71.4%) | 43 (70.5%) | 16 (55.2%) | 7 (58.3%) |
| The type and dosage of the drug to be used | 56 (88.9%) | 48 (78.7%) | 21 (72.4%) | 10 (83.3%) |
| Circumstances under which outpatient care would be possible | 52 (82.5%) | 44 (72.1%) | 18 (62.1%) | 6 (50%) |
| Conditions for inpatient admission | 55 (87.3%) | 56 (91.8%) | 23 (79.3%) | 10 (83.3%) |
| Question cannot be answered | 0 | 1 (1.6%) | 1 (3.4%) | 0 |
| **Need for further training in psychiatric emergencies/Need for further training for the PHEP as perceived by the PSs** | | | | |
|  | PHEP_inexp._  (n=6) | PS_inexp_ (n=61) | PS_experienced_  (n=29) | PS_very hexp._  (n=12) |
| Answered “Yes” | 6 (100%) | 58 (95.1%) | 29 (100%) | 9 (75%) |
|  | PHEP_exp_ (n=27) | PS_inexp_ (n=61) | PS_experienced_  (n=29) | PS_very hexp._  (n=12) |
|  | 19 (70.4%) | 58 (95.1%) | 29 (100%) | 9 (75%) |
|  | PHEP_very exp_ (n=63) | PSi_nexp_ (n=61) | PS_experienced_  (n=29) | PS_very hexp._  (n=12) |
|  | 49 (77.8%) | 58 (95.1%) | 29 (100%) | 9 (75%) |

**Supplementary table s1 summarizes the results all the questions of the questionnaires**. The questions from the questionnaires are shown as examples before the summarized response items of each question. Following from left to right are the responses of the emergency physicians and psychiatrists separately according to experience. Prehospital emergency physicians are categorized as follows: inexperienced: <1year additional qualification in emergency medicine, Experienced: 1–5 years of additional qualification in emergency medicine, very experienced: >5 years of additional qualification in emergency medicine. Psychiatrists are categorized according to their age: inexperienced: 25–35 years, Experienced: 35–45 years, Very experienced: >45 years. The responses of Prehospital emergency physicians and psychiatrists are presented in absolute values as well as percentages. Statistical differences were calculated by means of pairwise chi-square tests. PHEP = Prehospital emergency physician, PS = Psychiatrist, exp.: experienced. For further information we refer to “Schick B, Mayer B, Jäger M, Jungwirth B, Barth E, Eble M, Sponholz C, Muth CM, Schönfeldt-Lecuona C. Emergency medical care of patients with psychiatric disorders - challenges and opportunities: Results of a multicenter survey. BMC Emerg Med. 2022 Oct 28;22(1):173. doi: 10.1186/s12873-022-00722-5. PMID: 36303120; PMCID: PMC9615220.”

**Questionnaire for emergency physicians**

**1.**  As an emergency physician, you read the text " Emergency – psychiatric" on your pager.

What does that trigger in you? (Multiple answers possible)

|  | Sometimes I perceive psychiatric emergencies as pointless, so my intrinsic motivation can be quite low in such emergencies. |
| --- | --- |
|  | I frequently have the feeling that I am actually not sufficiently qualified to treat psychiatric patients. |
|  | I'm scared because I don't know what to expect in the situation. |
|  | I rate it as "neutral;" it's just an emergency like any other. |

You may also add further comments as free text.

**2.** Have you ever had the problem that you, as an emergency physician, indicated a patient for admission to psychiatry, but the on-site psychiatry department had to decline admission?

|  | Yes |  |  | No |
| --- | --- | --- | --- | --- |

**2 a.** If yes, what was the reason (multiple answers possible)?

|  | No available bed. |
| --- | --- |
|  | Patient is intoxicated and must first be monitored in the emergency room. |
|  | Emergency medical therapy (sedation/antipsychotics) impedes admission because the patient may need to be monitored closely. |
|  | Patient is not from the local psychiatric hospital's catchment area. |

You may also add further comments as free text

**3.** Please imagine the following scenario:

You are called as an emergency physician to a patient with post-traumatic stress disorder. When you arrive, the patient is agitated, hyperventilating and cannot be calmed down verbally. How do you proceed? (Multiple answers possible)

|  | I try to calm the patient verbally using the "talk down" approach. |
| --- | --- |
|  | I administer a benzodiazepine (e.g., midazolam/lorazepam) to the patient to calm them down. |
|  | I sedate the patient with a hypnotic (e.g., propofol) to calm them down. |
|  | I administer an antipsychotic (haloperidol) to calm the patient. |
|  | I ask the police for help in obtaining admission to the psychiatric ward. |
|  | I call the psychiatric department where the patient is already known and try to find a solution with my colleagues there over the phone. |
|  | In the end, I explain to the parents and friends present that the patient’s condition is improving on its own and, after consulting with the psychiatric department, I cancel the intervention. |

You may also specify further options that you consider suitable as free text.

**3 a.** Which type of medication application would you prefer in such a case?

|  | Intravenous administration. |
| --- | --- |
|  | Intraosseous administration. |
|  | MAD system (Mucosal Atomization Device) – "nasal atomizer." |
|  | Intramuscular administration. |
|  | Oral administration (if possible). |
|  | None of the above. |

**4.** Imagine the following alternative:

In the situation described above, you contact the psychiatrist by telephone. The psychiatrist tells you that a treatment concept for acute situations has been drawn up for the patient. Upon request, the patient's mother hands over the document to you. As an emergency physician, what information would you like to be included on such a treatment protocol? (Multiple answers possible)

|  | Possible symptoms (e.g., self-aggressive or aggressive towards others, screaming, crying, etc.) and expected progression. |
| --- | --- |
|  | Expected duration of the psychological emergency situation. |
|  | Supportive behavior (verbal reassurance, special caregiver, etc.). |
|  | Point at which medical intervention should be considered. |
|  | Type of medication and dosage that is usually sufficient – placebo use if appropriate, if previously agreed with the patient. |
|  | Outpatient care possible if the following criteria are met.... |
|  | Inpatient admission recommended if the following criteria are met.... |
|  | I cannot answer this question. |

You may also specify further options that you consider suitable as free text

**5.** Do you think a concept such as that outlined above would be appropriate in practice?

|  | Yes |  |  | No |
| --- | --- | --- | --- | --- |

**6.** Would you like more training on psychiatric emergencies for your emergency medicine practice, for example in the form of a one-day internship in an acute psychiatry setting?

|  | Yes |  |  | No |
| --- | --- | --- | --- | --- |

**7.** If yes, which topics would you consider particularly important in this context?

|  | extremely important | very important | relatively important | somewhat important | not at all important |
| --- | --- | --- | --- | --- | --- |
| Psychogenic seizure |  |  |  |  |  |
| Dealing with self-aggression and aggression towards others |  |  |  |  |  |
| Suicidality |  |  |  |  |  |
| Intoxication |  |  |  |  |  |
| Legal aspects of dealing with psychiatric patients |  |  |  |  |  |

You may also specify other important topics as free text.

In the questions below, we ask you to provide some information about yourself. The information you provide will not allow any conclusions to be drawn about your person.

**8.** How old are you?

|  | < 25 years |
| --- | --- |
|  | 25–35 years |
|  | 35–45 years |
|  | 45–55 years |
|  | 55–65 years |
|  | > 65 years |

**9.** Which medical discipline do you belong to (intended residency or specialist qualification)? (Multiple answers possible)

|  | Anesthesia |
| --- | --- |
|  | Internal medicine |
|  | Psychiatry |
|  | Neurology |
|  | Other discipline |

**10.** How many years have you held the additional designation of emergency medicine?

|  | < 1 year |
| --- | --- |
|  | 1–5 years |
|  | > 5 years |
|  | I do not have an additional qualification in emergency medicine |

**Questionnaire for psychiatrists**

(Schick B, Mayer B, Jäger M, Jungwirth B, Barth E, Eble M, et al. Emergency medical care of patients with psychiatric disorders - challenges and opportunities: Results of a multicenter survey. BMC Emerg Med. 2022;22:173.)

**1.** You are informed by your nurse that the emergency physician is on the phone and wants to admit a patient. What does this trigger in you? (Multiple answers possible).

|  | Sometimes I feel that the emergency physician's admission indication is flawed, and my motivation to care for patients admitted by emergency physicians is therefore diminished. |
| --- | --- |
|  | I frequently have the feeling that I am actually not sufficiently qualified to treat psychiatric patients. |
|  | I'm scared because I don't know what to expect in the situation. |
|  | I feel neutral about it; it's just a patient like any other. |

You may also add further comments as free text.

**2.** Have you ever had the problem that you, as a psychiatrist, had to reject an admission when the emergency physician indicated that the patient should be admitted to the psychiatric unit?

|  | Yes |  |  | No |
| --- | --- | --- | --- | --- |

**2a.** If yes, what was the reason (Multiple answers possible)

|  | No available bed. |
| --- | --- |
|  | Patient is intoxicated and must first be monitored in the emergency room. |
|  | Emergency medical therapy (sedation/antipsychotics) impedes admission because the patient potentially needs to be monitored closely. |
|  | Patient is not from the local psychiatric hospital's catchment area. |

You may also add further comments as free text

**3.** Please imagine the following scenario:

An emergency physician visits a patient with known post-traumatic stress disorder. Upon the arrival of the emergency physician, the patient is agitated, hyperventilating, and cannot be calmed verbally. What would you do, based on your psychiatric expertise? (Multiple answers possible)

|  | I try to calm the patient verbally using the "talk down" approach. |
| --- | --- |
|  | I administer a benzodiazepine (e.g., midazolam/lorazepam) to the patient to calm them down. |
|  | I sedate the patient with a hypnotic (e.g., propofol) to calm them down. |
|  | I administer an antipsychotic (haloperidol) to calm the patient. |
|  | I ask the police for help in obtaining an admission to the psychiatric ward. |
|  | I call the psychiatric department where the patient is already known and try to find a solution with my colleagues there over the phone. |
|  | In the end, I explain to the parents and friends present that the patient’s condition is improving on its own and, after consulting with the psychiatric department, I cancel the intervention. |

You may also specify further options that you consider suitable as free text.

**3 a.** Which type of medication application would you prefer in such a case?

|  | Intravenous administration. |
| --- | --- |
|  | Intraosseous administration. |
|  | MAD system (Mucosal Atomization Device) – "nasal atomizer." |
|  | Intramuscular administration. |
|  | Oral administration (if possible). |
|  | None of the above. |

**4.** Imagine the following alternative:

In the situation mentioned above, a treatment protocol for acute situations has been prepared for the patient, which the patient's mother hands over to the emergency physician. As a psychiatrist, what information would you want to communicate to the emergency physician by means of such a treatment protocol? (Multiple answers possible)

|  | Possible symptoms (e.g., self-aggressive or aggressive towards others, screaming, crying, etc.) and expected progression. |
| --- | --- |
|  | Expected duration of the psychological emergency situation. |
|  | Supportive behavior (verbal reassurance, special caregiver, etc.). |
|  | Point at which medical intervention should be considered. |
|  | Type of medication and dosage that is usually sufficient – placebo use if appropriate, if previously agreed with the patient. |
|  | Outpatient care possible if the following criteria are met.... |
|  | Inpatient admission recommended if the following criteria are met.... |
|  | I cannot answer this question. |

You may also specify further options that you consider suitable as free text

**5.** Do you think a concept such as that outlined above would be appropriate in practice?

|  | Yes |  |  |  | No |
| --- | --- | --- | --- | --- | --- |

**6.** Do you want a better insight into emergency medical care for your work as a psychiatrist, for example in the format of an optional one-day internship with an emergency medical response vehicle?

|  | Yes |  |  | No |
| --- | --- | --- | --- | --- |

**7.** Do you think that additional training on psychiatric emergencies is needed for colleagues practicing emergency medicine?

|  | Yes |  |  | No |
| --- | --- | --- | --- | --- |

**7a.** If yes, which topics would you consider particularly important in this context?

|  | extremely important | very important | relatively important | somewhat important | not at all important |
| --- | --- | --- | --- | --- | --- |
| Psychogenic seizure |  |  |  |  |  |
| Dealing with self-aggression and aggression towards others |  |  |  |  |  |
| Suicidality |  |  |  |  |  |
| Intoxication |  |  |  |  |  |
| Legal aspects of dealing with psychiatric patients |  |  |  |  |  |

You may also specify other important topics as free text.

In the questions below, we ask you to provide some information about yourself. The information you provide will not allow any conclusions to be drawn about your person.

**8.** How old are you?

|  | < 25 years |
| --- | --- |
|  | 25–35 years |
|  | 35–45 years |
|  | 45–55 years |
|  | 55–65 years |
|  | > 65 years |

**9.** Which medical discipline do you belong to (intended residency or specialist qualification)? (Multiple answers possible)

|  | Anesthesia |
| --- | --- |
|  | Internal medicine |
|  | Psychiatry |
|  | Neurology |
|  | Other discipline |

**10.** How many years have you held the additional designation of emergency medicine?

|  | < 1 year |
| --- | --- |
|  | 1–5 years |
|  | > 5 years |
|  | I do not have an additional qualification in emergency medicine |

| **Hospital** | **Emergency Missions with PHEP/year** | **Rescue helicopter Base** |
| --- | --- | --- |
| University hospital Jena | >5000 | X |
| Friedrichshafen Hospital | -- | X^1^ |
| University hospital Ulm | >6000 |  |
| University hospital Mannheim | >4600 | X² |
| Ulm military hospital | -- | X³ |

**sSupplementary table s2: Overview of the number of rescues per year (with or without helicopter) at participating hospitals.** ^1^1209 Rescue helicopter missions (2022), no further information about the emergency missions with PHEP per year available. ²1100 Rescue helicopter missions/year. ³1600 Rescue helicopter missions/year. Data are taken from and can be accessed through the clinics' websites. PHEP: Prehospital emergency physician.

| **Hospital** | **Number of beds for inpatient care** |
| --- | --- |
| University hospital Mannheim | 380 |
| Regional hospital Augsburg | 320 |
| Hospital Christophsbad Goeppingen | 102 |
| Center for Psychiatry Reichenau | 85 |
| Regional hospital Günzburg | 312 |
| KBO Lech-Mangfall-hospital Agatharied | 120 |
| Regional hospital Donauwörth (part of regional hospital Günzburg) | 18 |
| University hospital Ulm | 69 |
| University hospital Freiburg | 120 |
| University hospital Heidelberg | 250 |
| Danuvius hospital Pfaffenhofen | 80 |
| Regional hospital Kempten | 87 |

**Supplementary table s3: Overview of the number of beds for inpatient care at the participating hospitals (without number of outpatient beds).** Data are taken from and can be accessed through the clinics' websites.
